# The use of the WHO criteria to detect severe malaria among patients clinically diagnosed with uncomplicated malaria

**DOI:** 10.1371/journal.pgph.0003158

**Published:** 2024-08-15

**Authors:** Enoch Aninagyei, Richard Harry Asmah, Kwabena Obeng Duedu, John Gameli Deku, Kelvin Senyo Tanson, Yobo Mireku, Fred Gbadago, Desmond Omane Acheampong

**Affiliations:** 1 Department of Biomedical Sciences, School of Basic and Biomedical Sciences, University of Health and Allied Sciences, Ho, Volta Region, Ghana; 2 College of Life Sciences, Birmingham City University, City South Campus, Birmingham, United Kingdom; 3 Department of Medical Laboratory Sciences, School of Allied Health Sciences, University of Health and Allied Sciences, Ho, Volta Region, Ghana; 4 Laboratory Department, Ghana Health Service, Enyiresi Government Hospital, Enyiresi, Eastern Region, Ghana; 5 Laboratory Department, Ghana Health Service, Suhum Government Hospital, Suhum, Eastern Region, Ghana; 6 Department of Biomedical Sciences, School of Allied Health Sciences, University of Cape Coast, Cape Coast, Central Region, Ghana; Technical University of Kenya, KENYA

## Abstract

The World Health Organization (WHO) strict defining criteria were used to identify severe malaria among Ghanaian patients clinically diagnosed as uncomplicated malaria. From each study participant, blood haemoglobin (Hb) and plasma bilirubin levels were estimated using automated analyzers. According to the WHO, the criteria for diagnosing severe malaria among children (< 12 years) was assessed using Hb < 5 g/dL and among other patients ≥ 12 years, Hb < 7 g/dL with parasitemia > 10,000/μL, plasma bilirubin > 50 μmol/L amidst parasitemia > 100,000/μL and *P*. *falciparum* hyperparasitaemia (> 500,000 parasites/μL). Patients initially diagnosed with asymptomatic malaria (n = 347) were recruited. The parasitemia range was 540–863,402 parasite/μL. Overall, 86.2% of the patients had uncomplicated malaria while 13.8% of the patients were diagnosed with severe malaria of various origins. In children < 12 years, 10.8% (17/157) had Hb < 5g/dL with parasitaemia < 10,000 parasites/μL and in other patients (≥ 12 years), 6.3% (12/190) of them recorded Hb < 7g/dL with parasitaemia < 10,000 parasites/μL. Furthermore, 13.8% (48/347) had serum bilirubin levels > 50 μmol/L with parasitemia > 100,000/μL. In all the patients with hyperbilirubinemia, Hb levels fell below either 5g/dL or 7g/dL, for patients less than and 12 years or more, respectively. Finally, 1.7% (6/347) of the patients with malaria had parasite counts (> 500,000 parasites/μL). Irrespective of the etiology, patients diagnosed with severe malaria presented with pallor, vomiting, diarrhea, chills, fever and nausea, concurrently. Without comprehensive laboratory evaluation, patients with severe malaria could be misdiagnosed. Therefore, healthcare facilities need adequate human and logistical resources to be able to diagnose severe malaria for appropriate management to avert any untoward outcomes.

## Background

*Plasmodium falciparum* is the most common cause of malaria in Ghana [1]. Infection outcomes of *P*. *falciparum* are either asymptomatic [2] or clinical malaria [3]. Among patients with clinical malaria, cases could be presented as uncomplicated [4], complicated which is also referred to as severe malaria [5], or cerebral malaria [6]. The type of malaria and the degree of severity is influenced by the host immunity and the extent of unchecked parasite multiplication [7–9]. Uncomplicated malaria is mostly characterized by the absence of organ damage [10], but organ damage is common in severe malaria [11]. In both types of malaria, pallor, fever, anorexia, dyspepsia, epigastric discomfort, nausea, vomiting, and watery diarrhoea are evident. The only difference is that in severe malaria, these presentations are persistent while an irregular pattern is seen in the uncomplicated [5, 12]. Without prompt treatment, almost half of patients with severe malaria may die [11], while early detection and prompt treatment increase survival rate [13].

Malaria is still endemic in Ghana [14], with children under 5 years of age and pregnant women being the most vulnerable [15]. In 2022, the last nationwide survey for malaria put the average prevalence at 16.4%, with approximately 0.35 per 1000 deaths [16].

In clinical practice, most cases of clinical malaria, are managed as uncomplicated malaria. In resource-constrained areas, clinicians are trained to identity severe or complicated malaria when convulsions occur with positive malaria rapid diagnostic test outcome [5]. This could be misleading since other conditions may result in convulsion and severe malaria could occur without convulsion. Therefore, it is essential to properly classify all malaria cases before management, since in Ghana and elsewhere, management of uncomplicated malaria differs from severe malaria. Since 2004, the national antimalarial medicine policy adopted the use of oral artemisinin-combination therapy as the first and second line treatment options for uncomplicated malaria whereas all cases of severe malaria require hospitalization together with intramuscular (IM) or rectal artesunate or IM quinine [17].

To differentiate severe malaria from uncomplicated malaria, the World Health Organization (WHO) during its 2013 meeting, adopted strict defining criteria for severe malaria [18]. This study leveraged on routine laboratory analyses available in most districts hospitals in Ghana to assess malaria severity. Due to that, this study assessed severe malaria using the following criteria; (1) haemoglobin concentration < 5 g/dL in children < 12 years of age or < 7 g/dL in patients ≥ 12 years) together with a parasite count > 10,000/μL, (2) plasma or serum bilirubin > 50 μmol/L together with a parasite count > 100,000/μL and (3) hyperparasitaemia: *P*. *falciparum* parasitaemia > 500,000 parasites/μL or >10% parasitemia. These criteria were used to diagnose severe malaria because these tests are available in all district hospitals, polyclinics and some health centers in Ghana, where malaria is one of the top five diseases reported.

In 2022, a document published by the Ghana Ministry of Health and the Ghana Health Service reported that 93.1% of all malaria cases were classified as uncomplicated [19]. However, the document did not indicate the criteria to distinguish uncomplicated malaria from the other forms of malaria. Therefore, in this study, the WHO defining criteria were used to assess severe malaria in patients classified as having uncomplicated malaria.

## Methods

### Study design, study population, and study sites

A cross-sectional study design was employed in this study. Study participants were patients with clinical malaria whose malaria status was confirmed microscopically. Study participants were conveniently selected from three district hospitals, situated in malaria endemic districts in the Easter region of Ghana. The hospitals were Begoro District Hospital (Latitude: 6.3980637, Longitude: -0.3683076), Enyiresi District Hospital (Latitude: 6.43696, Longitude: -0.586211) and Suhum District Hospital (Latitude: 6.03714, Longitude: -0.4455). These hospitals were selected because the hospitals are located in forested part of the region where illegal mining and land tillage practices for crop farming have created conducive breeding sites for the malaria vector. Therefore, prevalence rates of malaria in the communities around the hospitals are expected to be high. The study participants were recruited from June–November 2023.

### Sample size calculation

The number of samples analysed in this study was determined based on the 2019 microscopy prevalence of malaria (32%) [20] in the Eastern region of Ghana. Using the formula, $N=\frac{Z^{2}p(1-p)}{d^{2}}$, where N is the sample size, z the confidence level at 95% (standard value of 1.96), p is the prevalence rate and d is the margin of error at 5%. The minimum sample size was calculated to be 334 participants.

### Inclusion and exclusion criteria

Participants included in this study were those aged 5 years and above. Participants above 18 years provided consent while parental consent was sought for participants less than 18 years, after which child assent was sought. Another inclusion criterion was being classified as having uncomplicated malaria, confirmed microscopically. However, participants with a history of sickle cell disease, HIV, hepatitis B, and C viruses, and those who received any parenteral fluids were excluded. Additionally, adult patients with uncontrolled hypertension and diabetes mellitus were excluded. Further, patients previously confirmed to have severe malaria, those on admission and pregnant women were excluded. Finally, participants who could not provide the required volume of blood were also excluded.

### Selection of participants

Due to the strict exclusion criteria, participants were conveniently recruited. Any patient that consented to take part in the study, and met the selection criteria was sampled. Participants were initially screened with CareStart (USA) malaria rapid diagnostic test (mRDT) kit, at the outpatient Department of the Hospital. Anyone found to be positive for *Plasmodium falciparum*-specific HRP2 was sent to the laboratory for microscopy confirmation. The mRDT was done as previously described [21].

### Blood sample collection and pre-analytical processing

Blood samples were collected by a trained Ghana Health Service phlebotomist. The area to be sampled (the antecubital fossa) was disinfected with 70% ethanol and allowed to air dry. Using a 23-gauge syringe, whole blood (approximately 4mL) was collected into an EDTA tube and mixed gently. The punctured side was dressed with cotton wool and covered with phlebotomy plaster. Haemoglobin estimation was done immediately after sample collection. Plasma was separated from the cells and kept at -20°C before the biochemical assays were done.

### Laboratory procedures

#### Establishment of active malaria status

Malaria microscopy was only done on samples that were found to contain *P*. *falciparum* specific HRP2 proteins. Microscopy was done using approximately 6 μL of blood. Dried blood smears were stained with 10% buffered-Giemsa stain for 10 minutes. Microscopy was done by two independent assessors, who were oblivious to the mRDT outcomes. Malaria parasites were quantified according to WHO protocol, as described elsewhere [3].

#### Determination of haemoglobin levels

In all facilities, the levels of haemoglobin were determined by Sysmex haematology analyser (Kobe, Japan). Well-mixed EDTA-anticoagulated whole blood was subjected to the analyser. Haemoglobin concentration was measured by cyanide-free colorimetric method. In-house quality control (QC) measures were employed to ensure the accuracy of the results. To ensure reliable, accurate and precise results from the three different analysers at the respective hospitals, the 1_3s_ Westgard rule [22] was used. Commercial QC samples (XN Check Low Level / Normal Level / High Level, Brooklyn, USA) were procured from DCL Laboratory Products, Ghana. The commercial QC samples were used to generate a Levey-Jenning chart, on which daily readings were plotted. Using the 1_3s_ Westgard rule, any QC reading outside the ±3s range violates this rule, hence, analysis will not be done till the anomaly is rectified.

#### Determination of plasma bilirubin levels

Bilirubin estimation was done by using the Biobase Biochemistry analyser (Guangzhou, China) and Elitech reagents obtained locally but manufactured by the ELITech Clinical Systems (France). The reagents were procured from DCL Laboratory Products, Ghana. Total bilirubin was estimated based on the Malloy-Evelyn modified, endpoint method. The accuracy and precision of the analyser were checked using the Elitrol I and II quality control commercial sera (ELITech Clinical Systems, France).

#### Diagnosis of severe malaria

Among the study participants with malaria, severe malaria was diagnosed using the following strict definitions. Having haemoglobin level < 5g/dL and < 7 g/dL amidst parasite count > 10,000 parasites/μL for children less than and 12 years and above, respectively. Separately, patients having plasma bilirubin levels > 50 μmol/L together with parasite count > 100,000 parasites/μL was diagnostic of severe malaria. Finally, patients with parasite count > 500,000 parasites/μL, irrespective of haemoglobin and bilirubin levels, was diagnostic of severe malaria. These defining criteria were recommended by the WHO for diagnosing severe malaria [18].

### Statistical analyses

All statistical analyses were performed using SPSS version 25 (IBM Corp, Armonk, NY). Descriptive statistics including frequencies, and percentages were used to summarize the demographic and clinical characteristics of the study population. Means and standard deviations for continuous variables were calculated and the geometric mean was utilized for parasite count since it tends to have a skewed distribution. Chi-square tests were used to assess associations between categorical variables. This included comparing proportions with severe malaria criteria across study sites, age groups, gender, and source of participants. It also included comparing clinical presentations, abstracted from participants’ clinical records, across the severe malaria criteria. The three criteria were used differently to define severe malaria among the study participants. The number of children less than or ≥ 12 years that had haemoglobin levels less than 5 g/dL or less than 7 g/dL, respectively, with parasite count > 10,000 parasites/μL was divided by the total number of children less than or 12 years and above to obtain the percentage of children or other patients that had severe malaria. On the other hand, the number of patients (of all ages) with plasma bilirubin levels > 50 μmol/L with parastaemia > 100,000 parasites/μL or parasitaemia > 500,000 parasites/μL was divided by the total number of the study participants to obain the percentage of patients who had severe malaria regarding the respective criteria. Statistical significance was defined as a two-tailed p-value < 0.05.

### Institutional review board statement

This study was reviewed and approved by the Ghana Health Service Ethics Review Committee (GHS-ERC 001/10/22). Written consent was sought from each participant and guardians of patients less than 18 years old. This work was carried out in accordance with The Code of Ethics of the World Medical Association (Declaration of Helsinki) for experiments involving humans. Participants were however, recruited anonymously.

## Results

### Demographic features of the study participants

At the end of the study period, blood samples were collected from 388 patients with uncomplicated malaria. Forty-one samples were rejected due to their unsuitability for hematological and biochemical analyses. The remaining 347 samples and their corresponding clinical information were further analyzed. Among the patients with valid samples, most of them were recruited from Begoro District Hospital 40.3% (140/347) and were within the aged group 5–14 years 40.9% (142/347). Additionally, the majority were females (62.8%), 43.5% under marital age (< 16 years), and minority (0.6%) of them were divorcees. Of the total, 3.7% had no formal education. Two hundred and thirty-two (232) of the patients were at various stages in their formal education, most of them were in pre-school while a minority (2.1%) of them were tertiary students. Finally, 102 of the patients had completed various levels of formal education; most of them (33.3%) being junior high school leavers. The frequencies of the other variables are shown in Table 1.

**Table 1:** Demographic characteristics of the study participants.

Variable	Frequency	Percent (%)
*Study sites*		
Begoro District Hospital	140	40.3
Enyiresi District Hospital	71	20.5
Suhum District hospital	136	39.2
*Age range (yrs)*		
5–14	142	40.9
15–24	89	25.6
25–34	38	11.0
35–44	24	6.9
45–54	14	4.0
> 54	40	11.5
*Gender*		
Male	129	37.2
Female	218	62.8
*Marital status*		
Under marital age (< 16 years)	151	43.5
Single	109	31.4
Married	74	21.3
Divorced	2	0.6
Widow/widower	11	3.2
*Highest education*		
No formal education	13	3.7
*Ongoing*		
Pre-school	99	28.5
Junior high	33	9.5
Primary	63	18.2
Senior high	34	9.8
Tertiary	3	0.9
*Completed*		
Primary	14	4.0
Junior high	34	9.8
Senior high	27	7.8
Tertiary	27	7.8

### Rates of severe malaria misdiagnosis among patients with uncomplicated malaria

Severe malaria was assessed using WHO defining criteria, as recently published (White, 2022). The defining criteria for severe malaria in this study were as follows.

1. Low hemoglobin level and parasite count > 10,000 parasites/μL (for children < 12 years of age, haemoglobin should be < 5g/dL and for patients ≥ 12 years, hemoglobin < 7g/dL).
2. For all age groups, plasma bilirubin > 50 μmol/L together with parasitemia ≥ 100,000 /μL.
3. Parasitemia > 10% or > 500,000/μL (hyperparasitemia).

Overall, 8.3% of the patients were misdiagnosed as uncomplicated malaria. Among the children (< 12 years), the rate was 10.8% (17/157) while in the patients ≥ 12 years with malaria, the rate was 6.3% (12/190) (x^2^ = 0.61, p = 0.687). Irrespective of the age of the patient, severe malaria was associated with the male gender. Using bilirubin as an indicator, 13.8% (48/347) of patients with severe malaria were misclassified. This criterion significantly identified more severe malaria cases by 5.4% (p = 0.039) than using haemoglobin as an indicator. Using bilirubin as an indicator, severe malaria was associated with children < 12 years (p = 0.011) and female gender (p = 0.026). Finally, using hyperparasitemia as an indicator, 1.7% (6/347) of patients with severe malaria were misclassified as uncomplicated malaria (Table 2).

**Table 2:** Factors associated with severe malaria among study variables.

Defining criteria		Hb < 5g/dL / parasitemia > 10,000/μL	Hb < 7 g/dL / parastemia > 10,000/μL	Plasma bilirubin > 50 μmol/L / parasitemia ≥ 100,000 /μL	Parasitemia > 500,000/μL
	Total	Children < 12 years	Patients ≥ 12 years		
*Study sites*					
Begoro District Hospital	140 (63, 77)^1^	6 (12.6%)	5 (6.4%)	20 (11.7%)	0 (0.0%)
Enyiresi District Hospital	71 (34, 37)^1^	3 (8.8%)	4 (10.8%)	9 (12.6%)	0 (0.0%)
Suhum District hospital	136 (60, 76)^1^	8 (11.6%)	3 (3.9%)	19 (13.9%)	6 (1.7%)
**p-value**		**0.564**	**0.422**	**0.836**	0.009*
*Age range* (years)					
< 12	157	Not applicable	Not applicable	31 (19.7)	5 (3.2%)
≥ 12	190	Not applicable	Not applicable	17 (8.9%)	1 (0.5%)
**p-value**		Not applicable	Not applicable	**0.011** *	**0.063**
*Gender*					
Male	129	11 (8.5%)	8 (6.2%)	10 (7.8%)	3 (2.3%)
Female	218	6 (2.8%)	4 (1.8%)	38 (17%)	3 (1.4%)
**p-value**		0.022*	0.038*	**0.026** *	**0.512**

^1^ (children < 12 years, other patients ≥ 12 years)

* Significant associations at p<0.05

### Clinical presentations associated with a particular class of severe malaria

The study participants with severe malaria exhibited 13 different signs and symptoms. The prevalence was as follows; pallor (57%, n = 198), vomiting (45.5%, n = 158), diarrhea (56.2%, n = 195), chills (67.4%, n = 234), and fever (78.9%, n = 274). Others were nausea (56.5%, n = 196), headache (60.2%, n = 209), fatigue (41.2%, n = 143), muscle ache (31.7%, n = 110), dizziness (1.7%, n = 6), anorexia (2.0%, n = 7), malaise (0.6%, n = 2) and abdominal pain (2.9%, n = 10). However, not all the WHO classified severe malaria cases presented with these signs and symptoms. Table 3 shows the malaria presentations that were presented by those who met the WHO classification for severe malaria. Patients with severe malaria, defined by low hemoglobin and high bilirubin levels, significantly presented with pallor, vomiting, diarrhea, fever, nausea, chills, and headache but not muscle aches. Finally, severe malaria is classified by hyperparasitemia associated with all symptoms except headache and muscle aches. From the foregoing, severe malaria due to low hemoglobin (< 7 g/dL), elevated bilirubin (≥ 50 μmol/L) and hyperparasitemia (> 500,000 parasites/μL) should be suspected in patients with malaria with multiple presentations of pallor, vomiting, diarrhea, chills, fever, nausea and headache.

**Table 3:** Clinical presentations in patients with severe malaria.

	Hb < 5g/dL / parasitemia > 10,000/μL		Hb ≤ 7 g/dL / parastemia > 10,000/μL		Plasma bilirubin > 50 μmol/L / parasitemia ≥ 100,000 /μL		Parasitemia > 500,000/μL	
Clinical presentations	Children < 12 years		Patients ≥ 12 years					
	Total (n = 17)	p-value	Total (n = 12)	p-value	Total (n = 48)	p-value	Total (n = 6)	p-value
Pallor (198)	14 (82.3%)	<0.001	8 (66.7%)	<0.001	41 (85.4%)	<0.001	5 (83.3%)	0.002
Vomiting (158)	14 (82.3%)	<0.001	7 (58.3%)	<0.001	46 (95.8%)	<0.001	4 (66.7%)	0.029
Diarrhoea (195)	11 (64.7%)	<0.001	7 (58.3%)	<0.001	38 (79.1%)	<0.001	4 (66.7%)	0.029
Chills (234)	17 (100%)	0.002	6 (50%)	0.009	40 (83.3%)	<0.001	6 (100%)	0.008
Fever (274)	15 (88.2%)	0.018	8 (66.7%)	<0.001	43 (89.6%)	0.004	6 (100%)	0.002
Nausea (196)	17 (100%)	<0.001	9 (75%)	<0.001	33 (68.8%)	<0.001	5 (83.3%)	0.002
Headache (209)	14 (82.3%)	<0.001	9 (75%)	<0.001	23 (47.9%)	<0.001	2 (33.3%)	0.174
Muscle ache (110)	9 (75%)	0.124	2 (16.7%)	0.104	14 (29.2%)	0.205	1 (16.7%)	0.425

p-values were generated by Chi-square test

## Discussion

To the best of our knowledge, this is the first time severe malaria is being assessed in patients with uncomplicated malaria in Ghana, using three World Health Organization (WHO) definitions. This study has shown that about 14% of patients with severe malaria were managed as uncomplicated malaria, which could have an unfavorable outcome on the patients. Using low hemoglobin as an indicator, 10.8% of the children less than 12 years old and 6.3% in patients 12 years old and over were found to have severe malaria. Elevated bilirubin and hyperparasitemia defining criteria also respectively identified severe malaria in 13.8% and 1.7% of the patients with malaria. Compared to patients ≥ 12 years, the prevalence of severe malaria, defined by very low hemoglobin, was about 2-fold higher in children. Previous studies have found that children were more vulnerable to severe forms of malaria probably because of relatively low or partial anti-malaria immunity compared to adult patients [23–25]. It is not surprising that mortality due to malaria is high in children less than 12 years [26]. The mean hemoglobin concentration of the children less than 12 years with malaria was 4.8 g/dL (19,060–693,490 parasites/μL) and that of patients 12 years or more was 6.9 g/dL (28,949–863,402 parasites/μL of blood). The very low anemia amidst parasitemia > 10,000 parasites/μL could be due to direct hemolysis induced by the parasites [23]. Further, the presence of parasites inside the red blood cells is known to reduce the deformability of the red cells. This phenomenon augments red cell hemolysis and clearance by the spleen [27]. Additionally, it has been established that *P*. *falciparum* hyperparasitemia promotes the elevation of serum tumor necrosis factor-alpha [28], which suppresses hematopoietic potential of the blood forming cells through marrow hypoplasia seen in acute malaria, and dyserythropoiesis, which results in ineffective erythropoiesis [29].

Bilirubin level above 50 μmol/L and parasitemia above 100,000 parasites/μL of blood was the second WHO criterion used to assess severe malaria. The criterion identified an overall prevalence of 13.8%. Patients 12 years or more with malaria and female patients with severe malaria were found to be significantly affected. Excessive production of bilirubin, especially the unconjugated form, has been attributed to the massive breakdown of red blood cells [30]. Hyperbilirubinemia is very common in malaria [10] and the direct relationship between malaria parasitemia and bilirubin levels have been revealed [31]. Parasitemia above 100,000 parasites/μL without bilirubin estimation cannot satisfy the severe malaria definition. Therefore, laboratory evaluation of bilirubin levels is essential to correctly classify severe malaria. It must, therefore, be noted that severe malaria with elevated serum bilirubin levels (>3 mg/dL or 50 μmol/L) and clinically evident yellow discoloration of the sclera and mucous membranes may indicate liver dysfunction [5]. Whereas, jaundice in severe malaria is primarily due to hemolysis of infected red blood cells [32], resulting in increased bilirubin production and impaired hepatic bilirubin clearance.

Finally, severe malaria defined by hyperparasitemia (parasitemia > 500,000 parasites/μL or >10% parasitemia) was 1.7%. Compared to the other defining criteria for severe malaria, severe malaria attributed to hyperparasitemia was low. This is explained by the fact that about 25–33% of the *P*. *falciparum* infected erythrocytes undergo cyto-adherence to vascular endothelium [33]. This reduces peripheral blood parasitemia [34], therefore, using peripheral blood may underestimate malaria parasitemia and accordingly underestimate severe malaria due to parasitemia. In low transmission areas, severe malaria may result with a parasite density of over 100,000 parasites/μL where malaria mortality could occur [35], while in high transmission areas, higher parasite densities may be tolerated [27]. Therefore, every malaria transmission zone should establish its own parasitemia cut-off range to define severe malaria. Columbia, for instance, has defined its cut-off parasitemia for severe malaria as > 50,000 parasites/μL of blood [35]. In spite of the foregoing, microscopy remains the gold standard for malaria diagnosis [36]. The microscopy technique is the only technique that is able to quantify the parasites in the peripheral blood when thick blood films are prepared [37]. Microscopy is also very useful when malaria rapid diagnostic test kits are unable to detect some parasites with histidine-rich protein 2 gene deletion [38]. When thin blood films are made, malaria parasite species identification becomes possible [39]. To be able to make the appropriate follow up after malaria treatment, microscopy plays a key role. This will help to suspect and confirm artemisinin resistance in resource limited settings in malaria endemic areas [40].

Several clinical presentations were recorded for patients with malaria. However, pallor, vomiting, diarrhea, chills, fever, and nausea were associated with all the defining criteria except headache which did not associate with hyperparasitemia. Therefore, this array of clinical presentations could be used to suspect severe malaria in patients with a parasite count of over 10,000 parasites/μL. Upon suspicion, laboratory confirmation is essential in such cases.

Severe malaria of any origin is a medical emergency [5], hence accurate classification of the disease and prompt treatment with the recommended therapy are recommended. This is essential to reduce associated mortality. In this study, 1.7–13.8% of the patients with severe malaria were managed as uncomplicated malaria. It is noteworthy that mortality due to severe malaria is very high and poor management could be a contributing factor. Misdiagnosis of severe malaria is likely to worsen the disease condition with its attendant fatality through coma, metabolic acidosis and anaemia [41, 42].

Previous works in Ghana that studied uncomplicated malaria were reviewed. In one of such studies, the prevalence of parasitemia ≥ 100,000/μL was 15.3% [43]. Without bilirubin assessment, patients with severe malaria were missed. A similar Ghanaian study on patients with malaria with parasite count > 100,000/ μL without bilirubin estimation was published [44]. Even though none of the mean hemoglobin levels fell below 7g/dL, hyperbilirubinemia amidst parasite count > 100,000/ μL was enough to diagnose severe malaria. Considering that fact that, in this study, the bilirubin indicator and parasitemia > 100,000/μL identified more patients with severe malaria than the low hemoglobin indicator. Taken together, hyperbilirubinemia and parasitemia >100,000/μL identified the most patients with severe malaria, compared to using low hemoglobin and hyperparasitemia. Therefore, the use of bilirubin > 50μmol/L together with parasitemia > 100,000/μL is the single most sensitive indicator for identification of severe malaria.

Due to logistics constraints, the assessment of severe malaria was limited to routine laboratory evaluation. Other laboratory procedures such as evaluation of metabolic acidosis, electrolytes levels, creatinine levels and blood glucose are other options. However, our laboratory lacks the capacity to measure bicarbonate ions and pH for assessment of metabolic acidosis. Secondly, because it was unethical to collect multiple samples from very sick children, separate samples were not collected blood glucose measurements.

Comprehensive assessment of patients for severe malaria is essential to inform the appropriate management options. Approximately one third of patients with severe malaria, especially, children have other conditions, known as Multiple Organ Dysfunction Syndrome (MODS) [5, 35, 45]. For this reason, other criteria for evaluating severe malaria should be incorporated into clinical and laboratory assessments. Aside the use of routine laboratory assessment, White, 2022 [5] has also suggested the use of the Glasgow Coma assessment, serum lactate measurement, hepatic dysfunction assessment, severe thrombocytopenia, metabolic acidosis and renal impairment assessment. These, undoubtedly, will not only provide an accurate and comprehensive assessment but also to ensure proper clinical management of severe malaria. Hypoglycemia, defined as blood glucose levels less than 40 mg/dL or 2.2 mmol/L in the absence of adequate oral intake [5] is one of the essential assessments for severe malaria. Occurrence of hypoglycemia in severe malaria may result from increased glucose consumption by parasitized red blood cells, impaired hepatic gluconeogenesis, and altered insulin secretion [46]. In fact, this can lead to neurological complications such as seizures and coma [47], if not promptly corrected.

### Limitations

This study had some limitations. Due to the cross-sectional study design employed in this study, the treatment outcome of the patients identified as having severe malaria was not found out. Additionally, it was unknown whether there was pre-existing anaemia or jaundice before the onset of the malaria. Further, for the purpose of dichotomous classification of malaria, cerebral malaria was not distinguished from severe malaria. Finally, other infectious or non-infectious diseases could confound the findings reported in this study.

## Conclusion

Up to 13.8% of patients with malaria assessed in this study presented with severe malaria. Clinical presentations associated with the severe forms of malaria were pallor, vomiting, diarrhea, chills, fever, nausea, and headache. Bilirubin levels above 50 μmol/L and parasitemia > 100,000 parasites/μL detected most of the patients with severe malaria. Therefore, it is recommended that bilirubin levels be measured together with parasite count for all patients with malaria. This will ensure the accurate diagnosis of severe malaria for proper disease management decisions to avert any untoward outcomes. That notwithstanding, malaria differential diagnoses, including influenza, dengue, and other arbovirus infections, must be emphasized as other causes of fever of unknown origin in endemic regions to avoid misdiagnosis and ensure prompt and appropriate treatment. To be able to comprehensively assess patients for severe malaria, healthcare facilities should be adequately equipped and resourced to provide both laboratory (blood glucose, lactate, bicarbonate, pH, creatinine, bilirubin, hemoglobin, platelet and parasite density) and clinical assessments (Glasgow coma, pulmonary and shock).

## References

[1] Owusu EDA, Brown CA, Grobusch MP, Mens P. Prevalence of Plasmodium falciparum and non-P. falciparum infections in a highland district in Ghana, and the influence of HIV and sickle cell disease. Malar J [Internet]. 2017 Dec 24;16(1):167. Available from: http://malariajournal.biomedcentral.com/articles/10.1186/s12936-017-1823-y. doi: 10.1186/s12936-017-1823-y 28438159 PMC5404324

[2] Bredu D, Donu D, Amoah LE. Dynamics of the Composition of Plasmodium Species Contained within Asymptomatic Malaria Infections in the Central Region of Ghana. Vatandoost H, editor. J Trop Med [Internet]. 2021 Feb 24;2021:1–7. Available from: https://www.hindawi.com/journals/jtm/2021/7419548/. 10.1155/2021/7419548 PMC7936885 33727937

[3] Aninagyei E. Repeated sampling improved the sensitivity of malaria microscopy in children under six years. BMC Res Notes [Internet]. 2020 Dec 4;13(1):508. Available from: https://bmcresnotes.biomedcentral.com/articles/10.1186/s13104-020-05359-w. doi: 10.1186/s13104-020-05359-w 33148309 PMC7640445

[4] Lwin KM, Ashley EA, Proux S, Silamut K, Nosten F, McGready R. Clinically uncomplicated Plasmodium falciparum malaria with high schizontaemia: A case report. Malar J [Internet]. 2008 Dec 11;7(1):57. Available from: https://malariajournal.biomedcentral.com/articles/10.1186/1475-2875-7-57. doi: 10.1186/1475-2875-7-57 18402713 PMC2365953

[5] White NJ. Severe malaria. Malar J [Internet]. 2022 Oct 6;21(1):284. Available from: https://malariajournal.biomedcentral.com/articles/10.1186/s12936-022-04301-8. doi: 10.1186/s12936-022-04301-8 36203155 PMC9536054

[6] Orish VN, Akake K, Lokpo SY, Kwadzokpui PK, Amegan-Aho KH, Mac-Ankrah L, et al. Evaluating the impact of COVID-19 pandemic on complicated malaria admissions and outcomes in the paediatric Ho Teaching Hospital of the Volta Region of Ghana. PLOS Glob Public Heal. 2022 Sep;2(9):e0000509. doi: 10.1371/journal.pgph.0000509 36962505 PMC10021415

[7] Langhorne J, Ndungu FM, Sponaas A-M, Marsh K. Immunity to malaria: more questions than answers. Nat Immunol [Internet]. 2008 Jul;9(7):725–32. Available from: https://www.nature.com/articles/ni.f.205. doi: 10.1038/ni.f.205 18563083

[8] Miller LH, Baruch DI, Marsh K, Doumbo OK. The pathogenic basis of malaria. Nature [Internet]. 2002 Feb;415(6872):673–9. Available from: https://www.nature.com/articles/415673a. doi: 10.1038/415673a 11832955

[9] Doolan DL, Dobaño C, Baird JK. Acquired Immunity to Malaria. Clin Microbiol Rev [Internet]. 2009 Jan;22(1):13–36. Available from: https://journals.asm.org/doi/10.1128/CMR.00025-08. 19136431 10.1128/CMR.00025-08 PMC2620631

[10] Aninagyei E, Tettey CO, Kwansa-Bentum H, Boakye AA, Ghartey-Kwansah G, Boye A, et al. Oxidative stress and associated clinical manifestations in malaria and sickle cell (HbSS) comorbidity. Agbor G, editor. PLoS One [Internet]. 2022 Jun 8;17(6):e0269720. Available from: https://dx.plos.org/10.1371/journal.pone.0269720. 35675349 10.1371/journal.pone.0269720 PMC9176834

[11] Dondorp AM, Fanello CI, Hendriksen IC, Gomes E, Seni A, Chhaganlal KD, et al. Artesunate versus quinine in the treatment of severe falciparum malaria in African children (AQUAMAT): an open-label, randomised trial. Lancet [Internet]. 2010 Nov;376(9753):1647–57. Available from: https://linkinghub.elsevier.com/retrieve/pii/S0140673610619241. doi: 10.1016/S0140-6736(10)61924-1 21062666 PMC3033534

[12] Khan M, Nisar H, Mushahid N. Clinical and Lab profile of severe and uncomplicated malaria: A prospective study from Khuzdar Balochistan. Pakistan J Med Sci [Internet]. 2021 Sep 9;37(7). Available from: https://pjms.org.pk/index.php/pjms/article/view/4210. doi: 10.12669/pjms.37.7.4210 34912418 PMC8613020

[13] Leopold SJ, Watson JA, Jeeyapant A, Simpson JA, Phu NH, Hien TT, et al. Investigating causal pathways in severe falciparum malaria: A pooled retrospective analysis of clinical studies. Beeson JG, editor. PLOS Med [Internet]. 2019 Aug 23;16(8):e1002858. Available from: https://dx.plos.org/10.1371/journal.pmed.1002858. 31442221 10.1371/journal.pmed.1002858 PMC6707545

[14] Tetteh JA, Djissem PE, Manyeh AK. Prevalence, trends and associated factors of malaria in the Shai-Osudoku District Hospital, Ghana. Malar J [Internet]. 2023 Apr 22;22(1):131. Available from: https://malariajournal.biomedcentral.com/articles/10.1186/s12936-023-04561-y. doi: 10.1186/s12936-023-04561-y 37087510 PMC10122813

[15] Ghana Health Service. Ghana malaria programme review final report. 2013.

[16] WHO. World malaria report 2023 [Internet]. 2023. Available from: https://www.who.int/publications/i/item/9789240086173.

[17] MOH G and N. Ministry of Health (MOH), Ghana Health Service (GHS) and National Malaria Control Programme (NMCP). 2020. Guidelines for Case Management of Malaria in Ghana, 4th Edition, 2020, Accra, Ghana.

[18] WHO. World Health Organization. Severe Malaria. Trop Med Int Health. 2014 Sept 19(1): 7–131. 10.1111/tmi.12313_2 25214480

[19] MOH/GHS. National malaria elimination strategic plan (NMESP) of Ghana: 2024–2028. 2023;

[20] Amoah LE, Donu D, Abuaku B, Ahorlu C, Arhinful D, Afari E, et al. Probing the composition of Plasmodium species contained in malaria infections in the Eastern region of Ghana. BMC Public Health [Internet]. 2019 Dec 2;19(1):1617. Available from: https://bmcpublichealth.biomedcentral.com/articles/10.1186/s12889-019-7989-1 31791319 10.1186/s12889-019-7989-1 PMC6889690

[21] Aninagyei E, Abraham J, Atiiga P, Antwi SD, Bamfo S, Acheampong DO. Evaluating the potential of using urine and saliva specimens for malaria diagnosis in suspected patients in Ghana. Malar J [Internet]. 2020 Dec 29;19(1):349. Available from: https://malariajournal.biomedcentral.com/articles/10.1186/s12936-020-03427-x 32993649 10.1186/s12936-020-03427-x PMC7526349

[22] Carroll TA, Pinnick HA, Carroll WE. Brief communication: Probability and the Westgard rules. Ann Clin Lab Sci. 2003;33(1):113–4. 12661907

[23] Cohee LM, Laufer MK. Malaria in Children. Pediatr Clin North Am [Internet]. 2017 Aug;64(4):851–66. Available from: https://linkinghub.elsevier.com/retrieve/pii/S0031395517300366 doi: 10.1016/j.pcl.2017.03.004 28734514 PMC5733786

[24] Ranjha R, Singh K, Baharia RK, Mohan M, Anvikar AR, Bharti PK. Age-specific malaria vulnerability and transmission reservoir among children. Glob Pediatr [Internet]. 2023 Dec;6:100085. Available from: https://linkinghub.elsevier.com/retrieve/pii/S2667009723000519 doi: 10.1016/j.gpeds.2023.100085 38440360 PMC10911094

[25] Mutsigiri-Murewanhema F, Mafaune PT, Shambira G, Juru T, Bangure D, Mungati M, et al. Factors associated with severe malaria among children below ten years in Mutasa and Nyanga districts, Zimbabwe, 2014–2015. Pan Afr Med J [Internet]. 2017;27. Available from: http://www.panafrican-med-journal.com/content/article/27/23/full/ doi: 10.11604/pamj.2017.27.23.10957 28761599 PMC5516661

[26] Schumacher R-F, Spinelli E. MALARIA IN CHILDREN. Mediterr J Hematol Infect Dis [Internet]. 2012 Nov 7;4(1):e2012073. Available from: http://www.mjhid.org/index.php/mjhid/article/view/2012.073 doi: 10.4084/MJHID.2012.073 23205261 PMC3507524

[27] White NJ. Malaria parasite clearance. Malar J [Internet]. 2017 Dec 23;16(1):88. Available from: http://malariajournal.biomedcentral.com/articles/10.1186/s12936-017-1731-1 28231817 10.1186/s12936-017-1731-1 PMC5324257

[28] Acheampong DO, Adu P, Ampomah P, Duedu KO, Aninagyei E. Immunological, haematological, and clinical attributes of rural and urban malaria: a case–control study in Ghana. J Parasit Dis [Internet]. 2021;45(3):806–16. Available from: doi: 10.1007/s12639-021-01363-4 34475663 PMC8368825

[29] Phillips RE, Pasvol G. Anaemia of Plasmodium falciparum malaria. Baillieres Clin Haematol [Internet]. 1992 Apr;5(2):315–30. Available from: https://linkinghub.elsevier.com/retrieve/pii/S0950353611800223 doi: 10.1016/s0950-3536(11)80022-3 1511178

[30] Creeden JF, Gordon DM, Stec DE, Hinds TD. Bilirubin as a metabolic hormone: the physiological relevance of low levels. Am J Physiol Metab [Internet]. 2021 Feb 1;320(2):E191–207. Available from: doi: 10.1152/ajpendo.00405.2020 33284088 PMC8260361

[31] Al-Salahy M, Shnawa B, Abed G, Mandour A, Al-Ezzi A. Parasitaemia and Its Relation to Hematological Parameters and Liver Function among Patients Malaria in Abs, Hajjah, Northwest Yemen. Interdiscip Perspect Infect Dis [Internet]. 2016;2016:1–5. Available from: http://www.hindawi.com/journals/ipid/2016/5954394/ doi: 10.1155/2016/5954394 27051422 PMC4804037

[32] Trampuz A, Jereb M, Muzlovic I, Prabhu RM. Clinical review: Severe malaria. Crit Care [Internet]. 2003 Aug;7(4):315–23. Available from: http://www.ncbi.nlm.nih.gov/pubmed/12930555 doi: 10.1186/cc2183 12930555 PMC270697

[33] Sherman IW, Eda S, Winograd E. Cytoadherence and sequestration in Plasmodium falciparum: defining the ties that bind. Microbes Infect [Internet]. 2003 Aug;5(10):897–909. Available from: https://linkinghub.elsevier.com/retrieve/pii/S128645790300162X doi: 10.1016/s1286-4579(03)00162-x 12919858

[34] Kingston HW, Ghose A, Plewes K, Ishioka H, Leopold SJ, Maude RJ, et al. Disease Severity and Effective Parasite Multiplication Rate in Falciparum Malaria. Open Forum Infect Dis [Internet]. 2017 Oct 1;4(4). Available from: https://academic.oup.com/ofid/article/doi/10.1093/ofid/ofx169/4767776 29302604 10.1093/ofid/ofx169 PMC5739038

[35] Padilla-Rodríguez JC, Olivera MJ, Guevara-García BD. Parasite density in severe malaria in Colombia. Carvalho LH, editor. PLoS One [Internet]. 2020 Jun 23;15(6):e0235119. Available from: https://dx.plos.org/10.1371/journal.pone.0235119 32574179 10.1371/journal.pone.0235119 PMC7310729

[36] CDC. Malaria Diagnostic Tests [Internet]. Atlanta, Georgia, United States; 2023. Available from: https://www.cdc.gov/malaria/diagnosis_treatment/diagnostic_tools.html

[37] Bejon P, Andrews L, Hunt-Cooke A, Sanderson F, Gilbert SC, Hill AV. Thick blood film examination for Plasmodium falciparum malaria has reduced sensitivity and underestimates parasite density. Malar J [Internet]. 2006 Dec 8;5(1):104. Available from: https://malariajournal.biomedcentral.com/articles/10.1186/1475-2875-5-104 17092336 10.1186/1475-2875-5-104 PMC1636647

[38] Aninagyei E, Smith-Graham S, Boye A, Egyir-Yawson A, Acheampong DO. Evaluating 18s-rRNA LAMP and selective whole genome amplification (sWGA) assay in detecting asymptomatic Plasmodium falciparum infections in blood donors. Malar J [Internet]. 2019;18(1):1–11. Available from: 10.1186/s12936-019-2850-7 31234871 PMC6591871

[39] Mathison BA, Pritt BS. Update on Malaria Diagnostics and Test Utilization. Kraft CS, editor. J Clin Microbiol [Internet]. 2017 Jul;55(7):2009–17. Available from: https://journals.asm.org/doi/10.1128/JCM.02562-16 28404673 10.1128/JCM.02562-16 PMC5483902

[40] Aninagyei E, Tetteh CD, Oppong M, Boye A, Acheampong DO. Efficacy of Artemether-Lumefantrine on various Plasmodium falciparum Kelch 13 and Pfmdr1 genes isolated in Ghana. Parasite Epidemiol Control [Internet]. 2020 Nov;11:e00190. Available from: https://linkinghub.elsevier.com/retrieve/pii/S2405673120300593 doi: 10.1016/j.parepi.2020.e00190 33163636 PMC7607505

[41] von Seidlein L, Olaosebikan R, Hendriksen ICE, Lee SJ, Adedoyin OT, Agbenyega T, et al. Predicting the Clinical Outcome of Severe Falciparum Malaria in African Children: Findings From a Large Randomized Trial. Clin Infect Dis [Internet]. 2012 Apr 15;54(8):1080–90. Available from: https://academic.oup.com/cid/article-lookup/doi/10.1093/cid/cis034 22412067 10.1093/cid/cis034 PMC3309889

[42] Idro R, Aloyo J, Mayende L, Bitarakwate E, John CC, Kivumbi GW. Severe malaria in children in areas with low, moderate and high transmission intensity in Uganda. Trop Med Int Heal [Internet]. 2006 Jan;11(1):115–24. Available from: https://onlinelibrary.wiley.com/doi/10.1111/j.1365-3156.2005.01518.x 16398762 10.1111/j.1365-3156.2005.01518.x

[43] Anyorigiya TA, Castel S, Mauff K, Atuguba F, Ogutu B, Oduro A, et al. Pharmacokinetic profile of amodiaquine and its active metabolite desethylamodiaquine in Ghanaian patients with uncomplicated falciparum malaria. Malar J [Internet]. 2021 Jan 6;20(1):18. Available from: https://malariajournal.biomedcentral.com/articles/10.1186/s12936-020-03553-6 33407454 10.1186/s12936-020-03553-6 PMC7788723

[44] Sakzabre D, Asiamah EA, Akorsu EE, Abaka-Yawson A, Dika ND, Kwasie DA, et al. Haematological Profile of Adults with Malaria Parasitaemia Visiting the Volta Regional Hospital, Ghana. Adv Hematol [Internet]. 2020 Feb 11;2020:1–6. Available from: https://www.hindawi.com/journals/ah/2020/9369758/ doi: 10.1155/2020/9369758 32095139 PMC7036090

[45] Bhutani A, Kaushik R, Kaushik R. A study on multi-organ dysfunction syndrome in malaria using sequential organ failure assessment score. Trop Parasitol [Internet]. 2020;10(2):86. Available from: http://www.tropicalparasitology.org/text.asp?2020/10/2/86/307781 doi: 10.4103/tp.TP_12_19 33747874 PMC7951073

[46] Tewari SG, Swift RP, Reifman J, Prigge ST, Wallqvist A. Metabolic alterations in the erythrocyte during blood-stage development of the malaria parasite. Malar J [Internet]. 2020 Dec 27;19(1):94. Available from: https://malariajournal.biomedcentral.com/articles/10.1186/s12936-020-03174-z 32103749 10.1186/s12936-020-03174-z PMC7045481

[47] Chianura L, Errante IC, Travi G, Rossotti R, Puoti M. Hyperglycemia in Severe Falciparum Malaria: A Case Report. Case Reports Crit Care [Internet]. 2012;2012:1–3. Available from: http://www.hindawi.com/journals/cricc/2012/312458/ doi: 10.1155/2012/312458 25161774 PMC4010067

